# Engineered Superinfective Pf Phage Prevents Dissemination of Pseudomonas aeruginosa in a Mouse Burn Model

**DOI:** 10.1128/mbio.00472-23

**Published:** 2023-04-11

**Authors:** Federico I. Prokopczuk, Hansol Im, Javier Campos-Gomez, Carlos J. Orihuela, Eriel Martínez

**Affiliations:** a Department of Microbiology, Heersink School of Medicine, University of Alabama at Birmingham, Birmingham, Alabama, USA; b Cystic Fibrosis Research Center, University of Alabama at Birmingham, Birmingham, Alabama, USA; Massachusetts General Hospital

**Keywords:** *Pseudomonas aeruginosa*, pathogenesis, filamentous phage, burn wounds, phage therapy, virulence, disseminated infection

## Abstract

Pf is a filamentous bacteriophage integrated in the chromosome of most clinical isolates of Pseudomonas aeruginosa. Under stress conditions, mutations occurring in the Pf genome result in the emergence of superinfective variants of Pf (SI-Pf) that are capable of circumventing phage immunity; therefore, SI-Pf can even infect Pf-lysogenized P. aeruginosa. Here, we identified specific mutations located between the repressor and the excisionase genes of Pf4 phage in the P. aeruginosa PAO1 strain that resulted in the emergence of SI-Pf. Based on these findings, we genetically engineered an SI-Pf (eSI-Pf) and tested it as a phage therapy tool for the treatment of life-threatening burn wound infections caused by PAO1. In validation experiments, eSI-Pf was able to infect PAO1 grown in a lawn as well as biofilms formed *in vitro* on polystyrene. eSI-Pf also infected PAO1 present in burned skin wounds on mice but was not capable of maintaining a sustained reduction in bacterial burden beyond 24 h. Despite not lowering bacterial burden in burned skin tissue, eSI-Pf treatment completely abolished the capability of P. aeruginosa to disseminate from the burn site to internal organs. Over the course of 10 days, this resulted in bacterial clearance and survival of all treated mice. We subsequently determined that eSI-Pf induced a small-colony variant of P. aeruginosa that was unable to disseminate systemically. This attenuated phenotype was due to profound changes in virulence determinant production and altered physiology. Our results suggest that eSI-Pf has potential as a phage therapy against highly recalcitrant antimicrobial-resistant P. aeruginosa infections of burn wounds.

## OBSERVATION

Pseudomonas aeruginosa is a major cause of acute and chronic infections, including those affecting the urinary tract, lung, and skin (1, 2). This opportunistic Gram-negative pathogen is a major health problem in nosocomial settings, as isolates are commonly multidrug resistant. For this reason, the World Health Organization has classified P. aeruginosa as a critical pathogen that urgently requires new therapeutic strategies (3). In this context, phage therapy has increasingly been used to treat recalcitrant P. aeruginosa infections, and the results so far have been encouraging (4, 5). However, these therapies, as in the case of antibiotics, also are a source of selective pressure and can result in the emergence of phage-resistant bacteria and, in turn, limit bacteria-phage interactions *in vivo* (6).

Pf is a filamentous bacteriophage that can be found integrated in the chromosome of most clinical isolates of P. aeruginosa (7); up to 68% of clinical strains of P. aeruginosa harbor a Pf phage (8). When grown *in vitro*, P. aeruginosa accumulates up to 10^11^ particles of Pf per milliliter of culture medium. Importantly, bacteria lysogenized with a filamentous phage are typically resistant to infection with the same phage type as a result of a mechanism called phage immunity. However, under stress conditions for the bacteria, a superinfective variant of Pf (SI-Pf) can emerge that has the capacity to circumvent phage immunity and superinfect already-Pf-lysogenized bacteria (9). Interestingly, a recent report by Tortuel et al. showed that P. aeruginosa strain PAO1 infected with the superinfective variant of Pf4 had reduced virulence in Belgian endive plant and Caenorhabditis elegans infection models (10). These observations led us to question whether lab-engineered SI-Pf has potential as a novel therapeutic strategy to treat humans with life-threatening infections of P. aeruginosa.

**FIG 1:**
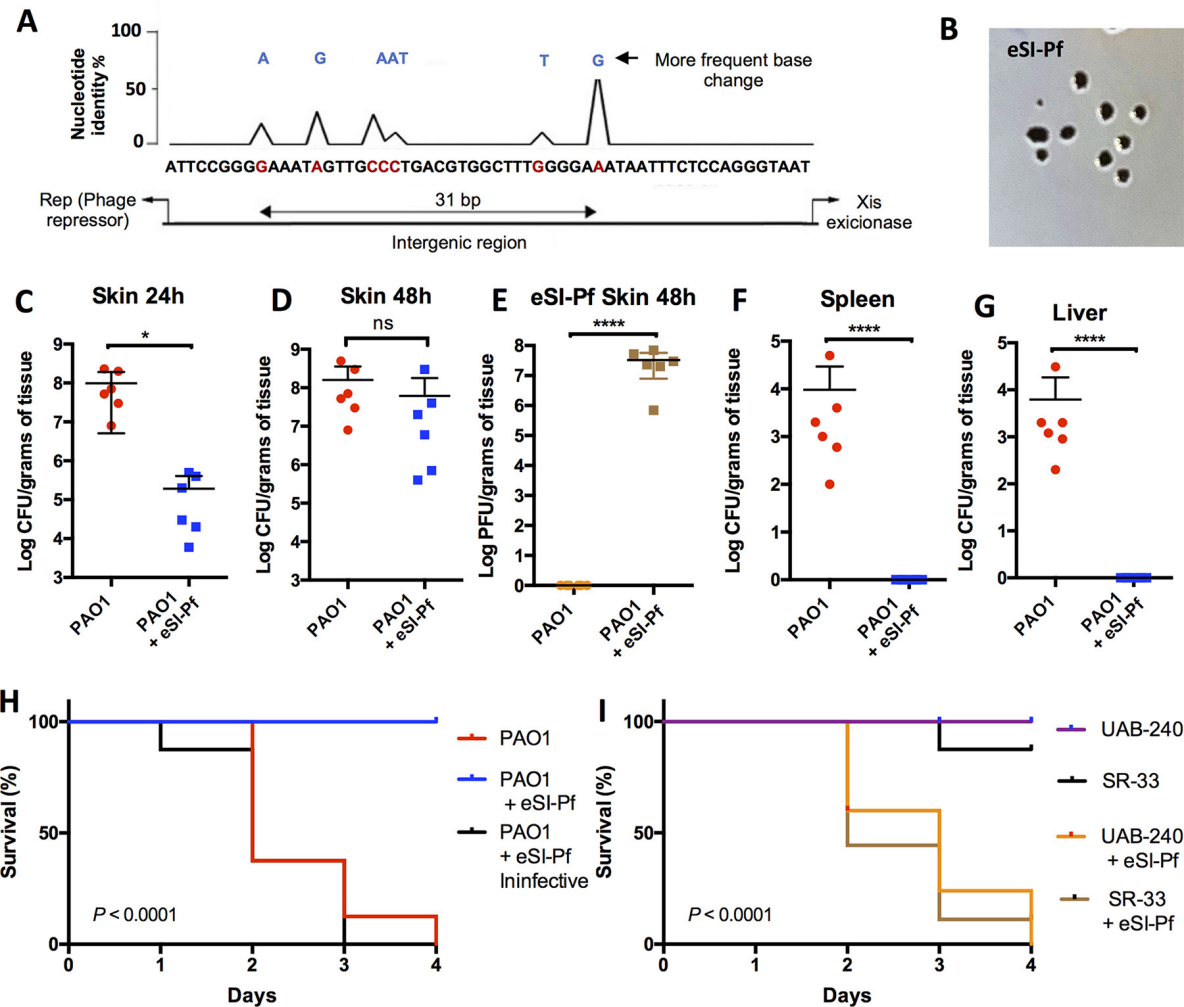
eSI-Pf leads to decreased P. aeruginosa virulence in a mouse burn model. (A) Sequence analysis of 40 SI-Pf individual clones isolated from single plaques. All mutations were localized in the intergenic region between the phage repressor and the excisionase genes. (B) eSI-Pf formed plaques on a PAO1 lawn. Image were obtained with a handheld camera. (C) Mice infected for 24 h with PAO1 were treated with eSI-Pf. At 24 h posttreatment, the skin showed reduced bacterial load compared to control. (D) However, no significant difference was detected 48 h posttreatment. (E) Mice with burn skin injury were infected with PAO1 and treated with eSI-Pf. The skin was homogenized 48 h postinfection and PFU were quantified by plotting on top of a PAO1 lawn. (F and G) PAO1 infected with eSI-Pf showed impaired dissemination to the spleen (F) and to the liver (G). (H) Kaplan Meier survival curves of mice infected with PAO1 and subsequently treated with eSI-Pf or a deactivated eSI-Pf control. (I) Kaplan Meier survival curves of mice infected with a clinical strain, UAB-240, or an environment isolate, SR-30, and subsequently treated with eSI-Pf. Student's *t* test was used to analyze data in panels C to G; a Mantel-Cox test was used for the data in panels H and I. Each dot is a biological replicate. Errors bars represent the standard errors of the means.

We first aimed to create an engineered version of Pf4 with the superinfective phenotype. It is known that the emergence of SI-Pf is due to mutations in the phage genome that result in excision from the bacterial chromosome with subsequent dysregulation of replication (11). However, the specific mutations identified so far have not been well delineated and have not been experimentally validated. We developed a genetic screen using the hypermutagenic strain Δ*mutS*, which produces SI-Pf at a high frequency (see Fig. S1A in the supplemental material) (12). Following sequencing, mutations associated with the formation of SI-Pf were determined to be localized at the intergenic region between the Pf repressor and the excisionase gene (Fig. 1A). Using PCR-based site-directed mutagenesis (see Text S1), we recreated the most abundant mutants into naive Pf, those with an Ala-to-Gly change at position 32 (A32G; 62.5%) or A6G (17.5%), in an attempt to recreate the SI phenotype (Fig. S1B). We also deleted the integration site (*attP*), the integrase, and other genes from the phage core (Fig. S1B). This was done to reduce the chances of an engineered superinfective variant reverting to wild type (WT) as well as to prevent its ability to integrate into the chromosome of the bacterium. This engineered phage, here referred to as eSI-Pf, produced plaques on a lawn of lysogenized PAO1, the key measure of the superinfective phenotype (13), that were also substantially larger than the plaques formed by naturally isolated SI-Pf (Fig. 1B). As our goal involved testing the ability of eSI-Pf to infect P. aeruginosa *in vitro* and *in vivo*, we also created a fluorescent version of eSI-Pf that encoded the tdTomato fluorescent protein (eSI-Pf-tdTomato), which formed red-ringed fluorescence plaques in PAO1 lawns in validation experiments (Fig. S1C).

10.1128/mbio.00472-23.5 TEXT S1 Materials and Methods. Download Text S1, DOCX file, 0.03 MB. Copyright © 2023 Prokopczuk et al. 2023 Prokopczuk et al. https://creativecommons.org/licenses/by/4.0/ This content is distributed under the terms of the Creative Commons Attribution 4.0 International license.

10.1128/mbio.00472-23.1 FIG S1 Construction of an engineered SI-Pf phage able to infect P. aeruginosa *in vitro* and *in vivo*. (A) Screening strategy to identify single mutations leading to the formation of the SI-Pf. Forty isolated colonies of the *mutS* mutant were cultured in LB broth, and the supernatant was used to infect a PAO1 lawn to select isolated plaques. (B) The most frequent mutations, A32xG and A6xG, were introduced in the replicative form of Pf4 phage. Additionally, all genes unnecessary for replication, assembly, and export of Pf, including the integrase-encoding gene, were deleted from the phage genome. (C) Fluorescent microscopy images of isolated plaques created by eSI-Pf-tdTomato on a PAO1 lawn. Scale bar, 100 μm. (D) Fluorescent microscopy image of a 24-h biofilm of PAO1 infected with eSI-Pf-tdTomato (red). Scale bar, 5 μm. (F) Fluorescent microscopy image of a burned mouse skin section infected with eSI-Pf-tdTomato. Scale bar, 50 μm. Download FIG S1, TIF file, 2.0 MB. Copyright © 2023 Prokopczuk et al. 2023 Prokopczuk et al. https://creativecommons.org/licenses/by/4.0/ This content is distributed under the terms of the Creative Commons Attribution 4.0 International license.

Using eSI-Pf-tdTomato, we determined the superinfective phage was capable of infecting established biofilms: 30 to 35% of all bacteria in a 24-h PAO1 biofilm superinfected with eSI-Pf-tdTomato fluoresced red (Fig. S1D). eSI-Pf could also infect P. aeruginosa *in vivo* under clinically relevant conditions, as fluorescence microscopy analysis of PAO1-infected burn skin samples from mice that had been administered eSI-Pf-tdTomato showed that that the phage successfully infected bacteria present in the wound site (Fig. S1E). Once we confirmed that eSI-Pf could infect P. aeruginosa *in vivo*, we tested its efficacy against P. aeruginosa. PAO1-infected, burned mice treated with 10^8^ particles of eSI-Pf showed a significant reduction in bacterial load 24 h posttreatment (Fig. 1C). However, at 48 h we determined that the bacterial load had recovered (Fig. 1D). Notably, and despite this recovery in burden, we observed a high prevalence of eSI-Pf in the burn sites of treated mice (Fig. 1E) and impaired ability of P. aeruginosa to disseminate from the skin to internal organs (Fig. 1F and G). In contrast, mice that did not receive eSI-Pf treatment had up to 5.0 × 10^4^ CFU/g of tissue in the spleen and similar levels in the liver (Fig. 1F and G). All eSI-Pf-treated mice fared better in subsequent days (see Video S1) and survived burn infection, whereas all untreated mice died (Fig. 1H). Importantly, protection was not observed when mice were treated with eSI-Pf that had been deactivated by boiling for 10 min before infection (Fig. 1H). A similar result was observed when burned mice were infected with a clinical strain, UAB-240, or an environmentally isolated strain, SR-33, and treated with eSI-Pf (Fig. 1I).

**FIG 2:**
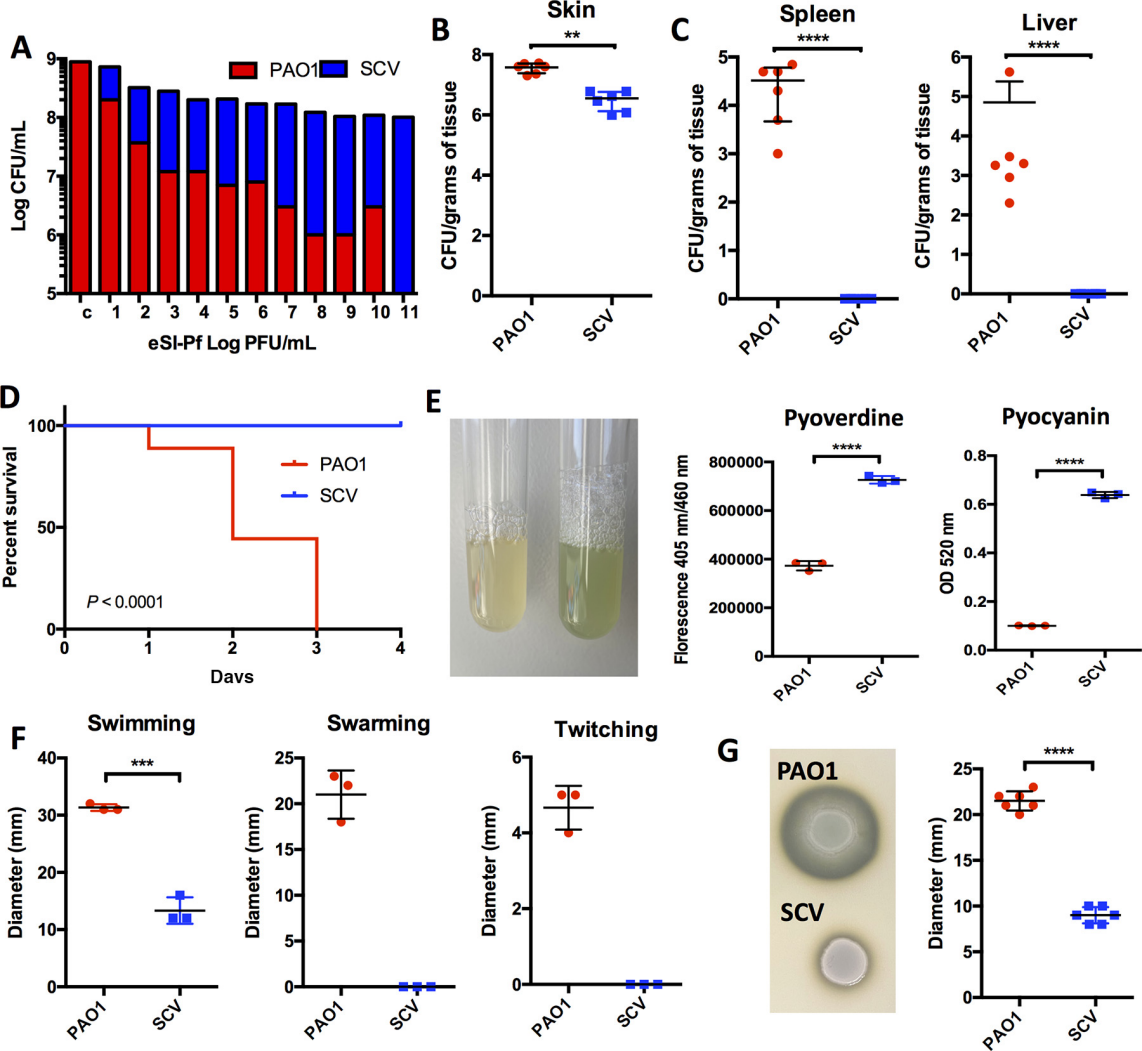
eSI-Pf infection induces formation of an attenuated small-colony variant. (A) eSI-Pf infection of P. aeruginosa induced formation of the SCV. This occurred in a phage concentration-dependent manner. (B) Mice with a burn skin injury were infected with SCV. The SCV was able to colonize the injury site but did so less efficiently than PAO1 WT. (C) The SCV showed impaired dissemination to the spleen and the liver. (D) Kaplan Meier survival curves of mice infected with the SCV versus PAO1 WT showed that the SCV is attenuated in the mouse burn model. (E) The SCV overproduced pyoverdine and pyocyanin pigments *in vitro.* (F and G) The SCV showed reduced motility (F) and protease secretion (G) compared to the PAO1 WT. Student's *t* test was used to analyze data in panels B, C, and E to G; a Mantel-Cox test was used for the panel D data. Each dot is a biological replicate. Errors bars represent the standard errors of the means.

Since eSI-Pf treatment did not result in a sustained reduction in bacterial load at 48 h, we considered the possibility that P. aeruginosa that survived eSI-Pf challenge became attenuated and was therefore unable to disseminate. Previous studies had shown that superinfective phage production correlates with the emergence of a small-colony variant (SCV) phenotype of P. aeruginosa (9). In agreement, infection of PAO1 with eSI-Pf resulted in formation of an SCV *in vitro* (Fig. S2A). The conversion of PAO into an SCV was proportional with the eSI-Pf concentration used to infect the monolayer (Fig. 2A). Importantly, the SCV isolated from an eSI-PF-infected lawn of PAO1 colonized the skin of burned mice less efficiently than WT PAO1 (Fig. 2B). Moreover, we did not detect dissemination of the SCV to internal organs (Fig. 2C). Finally, all mice treated with the SCV survived the infection (Fig. 2D). Interestingly, the SI-Pf-derived SCV showed pigment dysregulation on plates (Fig. S2B). Quantification of pyoverdine and pyocyanin revealed the overproduction of both pigments by the SCV (Fig. 2E). We also observed that the SCV had impaired motility (Fig. 2F) and reduced protease production (Fig. 2G) (14). Together, these observations suggested that eSI-Pf imposes considerable virulence gene dysregulation on the bacterium, and this helps to explain the attenuated phenotype of the SCV *in vivo*. Interestingly, the SCV also showed increased susceptibility to several classes of antibiotic, supporting the notion of a phage-caused dysregulated physiology and, moreover, its feasibility for a phage-antibiotic combinatorial therapy in a P. aeruginosa-infected individual (Fig. S3).

10.1128/mbio.00472-23.2 FIG S2 Isolation of the SCV from a culture of PAO1 treated with eSI-Pf. (A, left) Untreated PAO1; (right) eSI-Pf-treated PAO1. The arrow indicates a representative SCV. (B) Isolated colonies of PAO1 and the SCV were cultured overnight in tryptic soy broth. The SCV shows dysregulation of pigment production. Download FIG S2, TIF file, 1.8 MB. Copyright © 2023 Prokopczuk et al. 2023 Prokopczuk et al. https://creativecommons.org/licenses/by/4.0/ This content is distributed under the terms of the Creative Commons Attribution 4.0 International license.

10.1128/mbio.00472-23.3 FIG S3 SCVs have increased sensitivity to antibiotics. PAO1 and the SCV were treated with several families of antibiotics: chloramphenicol, ampicillin, rifampin, and erythromycin. Bacteria were incubated overnight with the antibiotics, and viability was determined by optical cell density at 600 nm. Download FIG S3, TIF file, 0.5 MB. Copyright © 2023 Prokopczuk et al. 2023 Prokopczuk et al. https://creativecommons.org/licenses/by/4.0/ This content is distributed under the terms of the Creative Commons Attribution 4.0 International license.

In summary, our observations suggest that the superinfective variant of the filamentous phage Pf has meaningful potential as a new therapeutic weapon to prevent bacterial dissemination in burn patients infected with P. aeruginosa. This therapeutic strategy should, however, be approached with considerable caution and more study, as the WT version of Pf is involved in several aspects of P. aeruginosa’s pathogenesis, and our study covered only a small area of possibility.

10.1128/mbio.00472-23.4 VIDEO S1 eSI-Pf-treated mice with P. aeruginosa-infected burn wounds were more alert and responsive than controls. Burned mice were infected with PAO1. Four hours postinfection, a cohort of mice was treated intradermally with eSI-Pf at the burn infection site. Videos were taken at 24 h postinfection. (Top) Treated with eSI-Pf; (bottom) untreated control. Download Movie S1, MP4 file, 1.6 MB. Copyright © 2023 Prokopczuk et al. 2023 Prokopczuk et al. https://creativecommons.org/licenses/by/4.0/ This content is distributed under the terms of the Creative Commons Attribution 4.0 International license.
