# Supplementary material for: Engineered Superinfective Pf Phage Prevents Dissemination of Pseudomonas aeruginosa in a Mouse Burn Model
Source: mBio. 2023 Apr 11;14(3):e00472-23. doi: 10.1128/mbio.00472-23 (PMC10294672; doi:10.1128/mbio.00472-23)
Supplement: TEXT S1 [file mbio.00472-23-s0001.docx]

**Supplemental Material and Methods**

**Strains and culture conditions.** P. aeruginosa strain PAO1 (obtained from the Manoil Lab at the University of Washington, Seattle, WA, USA) (1) and its isogenic mutant Δ*mutS* (2) were used in this study. The strains were routinely grown in LB medium, or LB containing agar when solid medium was required. Swimming, swarming and twitching motilities were studied using the methods described by Rashid and Kornberg (3).

**DNA isolation and manipulation.** Plasmid DNA was prepared using a QIAprep Spin Miniprep kit (Qiagen), which was used also to purify the replicative form of Pf phage. Q5 polymerase and modification enzymes were used according to the manufacturer’s instructions (NE Biolabs). The DNA was visualized with ethidium bromide (1 µg/ml). The replicative forms of the SI-Pf variants were used for sequencing using new generation sequencing (NGS). The sequences were analyzed using SnapGene 5.2.4.

**Production and isolation of SI-Pf variants.**A *mutS*-deficient strain which spontaneously produces superinfective Pf (SI-Pf) viral particles was used as the SI-Pf donor. Isolated colonies of Δ*mutS* were grown in 5 ml of LB for 20 h with shaking (240 rpm) at 37°C. One milliliter of the culture was spun down, and the supernatant was serially filtered through 0.45- and 0.22-µm-pore-size filters (sartorius). Cell-free supernatants containing SI-Pf particles were used to produce isolate phage plaques on a lawn of PAO1 lawn.

**Construction and purification of the engineered SI-Pf phage.** Site-direct mutagenesis in Pf phage and fragment deletions were performed by inverse PCR using the replicative from of Pf phage and the primers; Pf-RV-NotI: GACAGCGGCCGCGTCAGCTTCATTTCTTGCCTTCCA and Pf-FW-NotI: GACAGCGGCCGCCAGGTCCTTTGCCACCAACC. For site-direct mutagenesis, A32G was created using primers with mutagenic ends; A32G-FW: GATAATTTCTCCAGGGTAATTATTTCTCTAGC; A32G-RV: TCCCCAAAGCCACGTCAG; A6G-FW: GGTTGCCCTGACGTGGC and A6G-RV: ATTTCCCCGGAATAAATTTCTATATGAGCAC. To create a SI-Pf version expressing tdTomato we digested PUC-tdTomato vector with notI and the fragment containing the tdTomato gene was cloned into the replicative form of the eSI-Pf. The supernatant of transformed PAO1 was analyzed for red plaque formation a PAO lawn. To purify eSI-Pf, the supernatant of PAO1 infected with the eSI-Pf was serially filtered through 0.45- and 0.22-µm-pore-size filters (sartorius). Phage particles were precipitated from the filtered supernatant by adding sodium chloride and polyethylene glycol to give final concentrations of 3 and 5% (wt/vol), respectively. The mixture was incubated on ice for 30 min and centrifuged at 12,000 × *g* for 20 min. The supernatant was discarded, and the phage-containing pellet was resuspended in 1 ml of phosphate-buffered saline.

**Phage titration.** Titers of SI-Pf were routinely determined by dropping serial dilutions of filtered culture supernatants of phage-producing cells or purified particles onto soft LB agar (0.4%) plates containing the PAO1 strain. The numbers of plaques were determined after overnight incubation at 37°C. Isolated plaques were used to isolate monoclonal SI-Pf when required.

**Pigment quantification.** PAO1 and the SCVs were grown overnight in LB broth at 37 °C. The cultures were centrifuged, and the supernatant was filtered through a 0.22 membrane filter (Corning). For Pyoverdine extraction, ∼900 μL of the supernatant was filled to 2 mL with HPLC grade chloroform (Fisher Scientific), mixed thoroughly and centrifuged for 5 min at 10,000 rpm. Pyoverdine in the aqueous phase was measured by fluorescence (λEX = 405 nm and λEM = 460 nm) using a microplate reader (BioTek). For pyocyanin, the supernatant was extracted with 3 mL of chloroform and then re-extracted into 1 mL of 0.2 N HCl. Absorbance was measure at 520 nm.

**Quantification of biofilm formation.** Biofilm assays were performed following the O'Toole protocol (4). Briefly, P. aeruginosa strains were cultured overnight in LB agar plates at 37 °C. Bacterial suspensions were prepared in M63 medium to an OD_600_=1. Ten microliters of bacterial suspension were inoculated into each well of a 96-well microtiter plate containing 200 μl of M63 complete media. Biofilms were allowed to form at 30 °C overnight. For biofilm quantification the wells were washed two times with PBS and 200 μl of 0.1% crystal violet was added to the wells and incubated for 10 min. Wells were washed three times with phosphate-buffered saline, then crystal violet-stained biofilm was solubilized with 250 μl of 30% acetic acid and the absorbance was measured at 550 nm.

**Fluorescence microscopy.** Visualization of the biofilm and phage plaques were conducted using Leica LMD 6 and Nikon eclipse Ti microscope. To make the biofilm structure, we directly used slide glass as surface and made the biofilm for 48 hours incubation. Phage was inoculated at the 24 hours timepoint. The fluorescence intensity was compared using red fluorescence (tdTomate-SI-Pf). For the plaque images, overnight culture of SI-Pf on PAO1 was used.

**Back burn mice infection.** Mice infection was performed as previously described (5). Briefly, five- to six-week-old BALBc mice (Jackson Labs) were anesthetized with a mixture of xylazine and ketamine. The back was shaved with an electric clipper and then depilated with depilatory cream. The burn was induced using a hot bar and the infection was performed intradermally with 100 μl of bacterial suspension containing 10^6^ CFU of *P. aeruginosa* PAO1 or the small colony variant (SCV). The bacterial burden in the skin, liver and spleen was assessed by homogenizing the organ and plating dilutions on LB agar plates.

**Treatment of burn wound infection with eSI-Pf.**Four hours post-infected mice were treated intradermally with 100μl of purified eSI-Pf containing 10^7^ PFU of eSI-Pf or 100 μl of phosphate buffer saline for controls. After treatment mice were monitored each day for symptoms and deaths for 10 days or euthanized 24 or 48 hours postinfection to determine bacterial burden in the skin, liver and spleen.

**Ethics statement.** The animal experimental design was approved by the Institutional Animal Care and Use Committee at The University of Alabama at Birmingham, UAB (protocol no. IACUC-22197).

**Statistical analysis.** Kaplan-Meier plots of mice survival experiments were compared using the log-rank (Mantel-Cox) test. We used 6 mice per condition. In the rest of the experiments one-way ANOVA or unpaired *t*-test was used. All statistical analyses were performed using GraphPad Prism 8 software (GraphPad Software, La Jolla, CA).

**Supplemental Material and Methods References**

1. Jacobs MA, Alwood A, Thaipisuttikul I, Spencer D, Haugen E, Ernst S, Will O, Kaul R, Raymond C, Levy R, Chun-Rong L, Guenthner D, Bovee D, Olson MV, Manoil C. 2003. Comprehensive transposon mutant library of Pseudomonas aeruginosa. Proc Natl Acad Sci U S A 100:14339-44.

2. Martinez E, Campos-Gomez J. 2016. Pf Filamentous Phage Requires UvrD for Replication in Pseudomonas aeruginosa. mSphere 1.

3. Rashid MH, Kornberg A. 2000. Inorganic polyphosphate is needed for swimming, swarming, and twitching motilities of Pseudomonas aeruginosa. Proc Natl Acad Sci U S A 97:4885-90.

4. O'Toole GA. 2011. Microtiter dish biofilm formation assay. J Vis Exp doi:10.3791/2437.

5. Maura D, Bandyopadhaya A, Rahme LG. 2018. Animal Models for Pseudomonas aeruginosa Quorum Sensing Studies. Methods Mol Biol 1673:227-241.
