# Supplementary figures and images for: Engineered Superinfective Pf Phage Prevents Dissemination of Pseudomonas aeruginosa in a Mouse Burn Model
Source: mBio. 2023 Apr 11;14(3):e00472-23. doi: 10.1128/mbio.00472-23 (PMC10294672; doi:10.1128/mbio.00472-23)

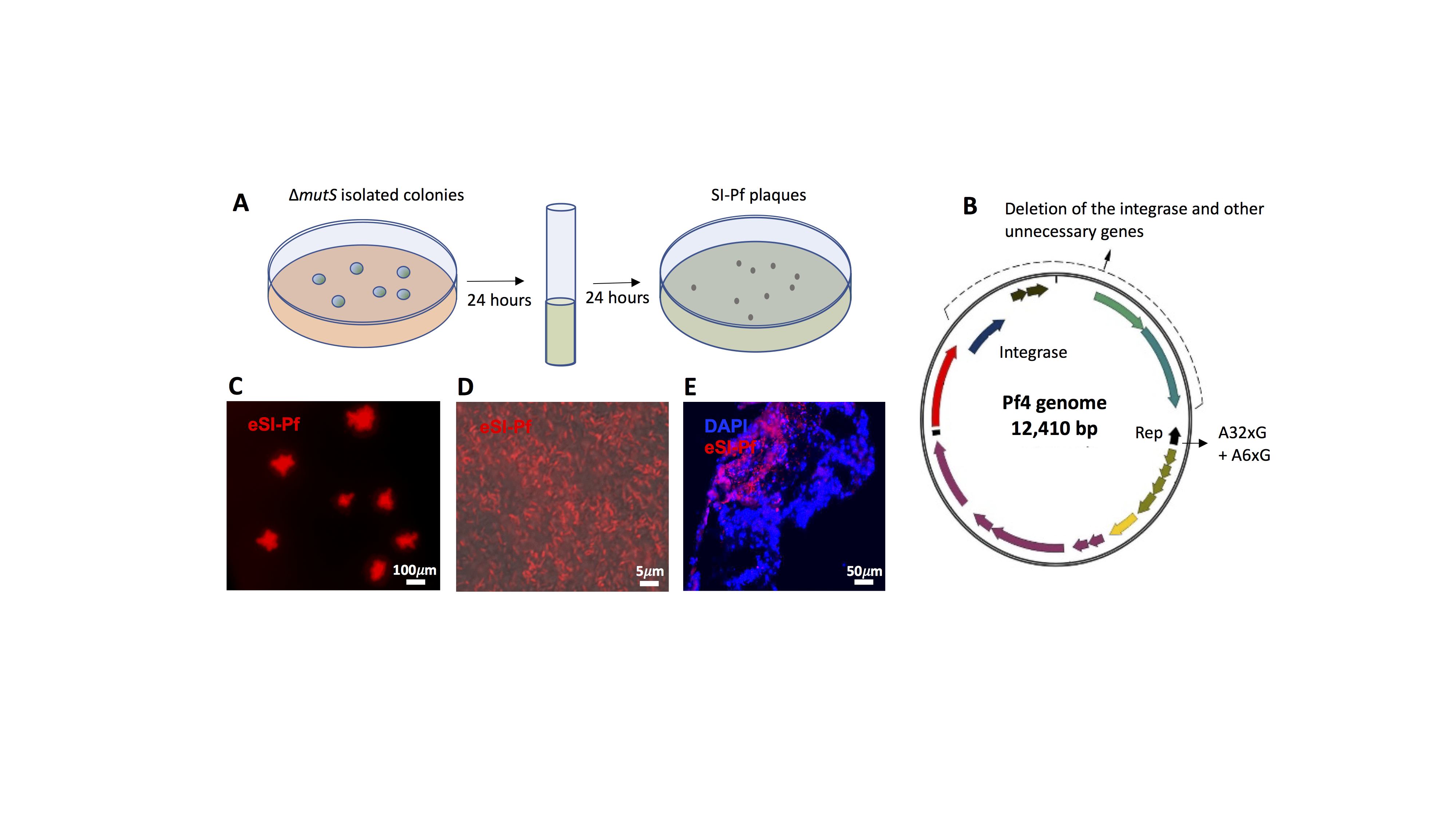

Supplement: FIG S1 [file mbio.00472-23-s0002.tif]

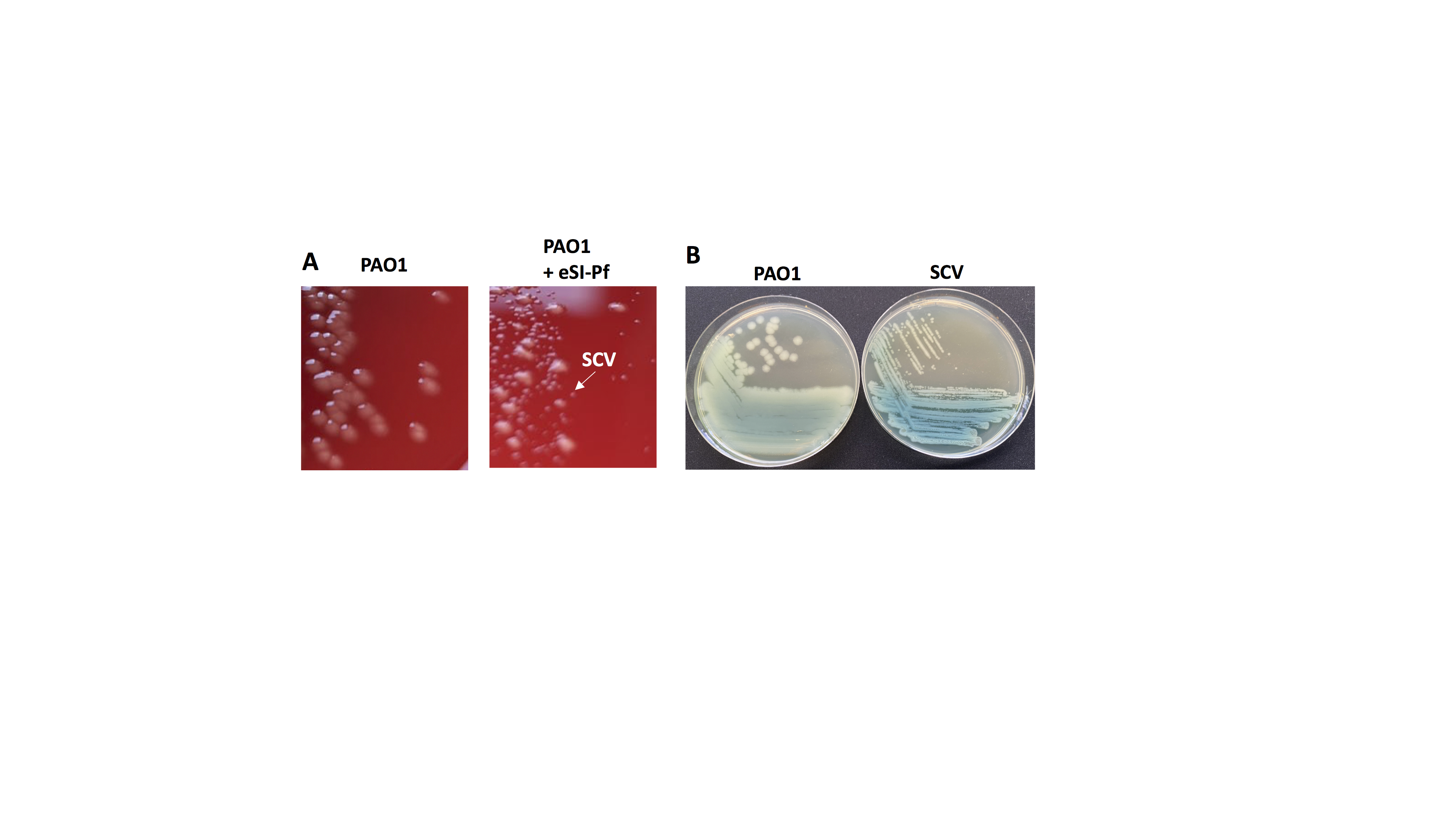

Supplement: FIG S2 [file mbio.00472-23-s0003.tif]

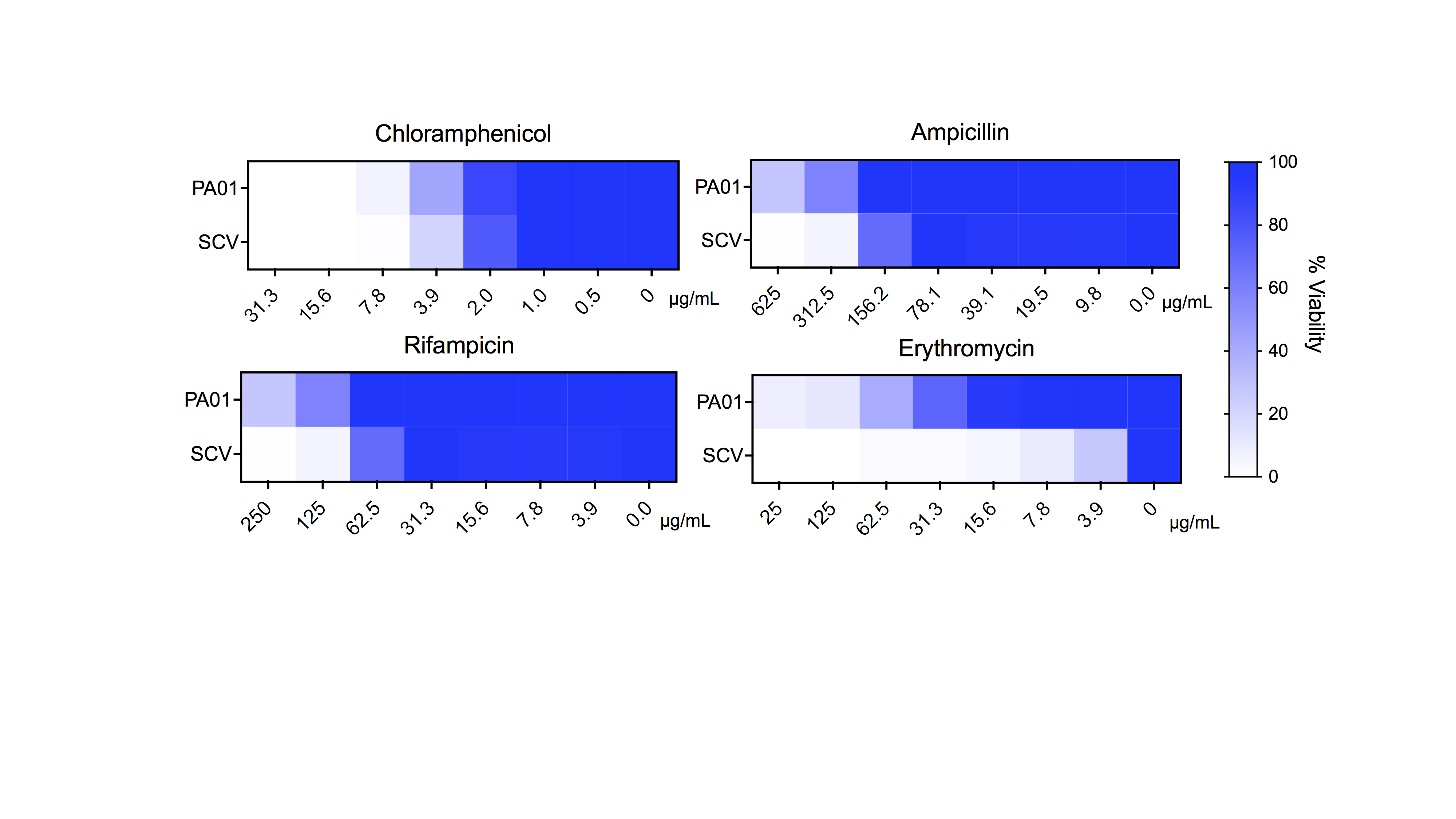

Supplement: FIG S3 [file mbio.00472-23-s0004.tif]
